# Improved mitochondrial function in salmon (*Salmo salar*) following high temperature acclimation suggests that there are cracks in the proverbial ‘ceiling’

**DOI:** 10.1038/s41598-020-78519-4

**Published:** 2020-12-10

**Authors:** Lucie Gerber, Kathy A. Clow, Felix C. Mark, Anthony K. Gamperl

**Affiliations:** 1 Department of Ocean Sciences, Memorial University, St. John’s, NL Canada; 2 Section Integrative Ecophysiology, Alfred Wegener Institute, Helmholtz Centre for Polar and Marine Research, Bremerhaven, Germany

**Keywords:** Cardiovascular biology, Metabolism, Respiration

## Abstract

Mitochondrial function can provide key insights into how fish will respond to climate change, due to its important role in heart performance, energy metabolism and oxidative stress. However, whether warm acclimation can maintain or improve the energetic status of the fish heart when exposed to short-term heat stress is not well understood. We acclimated Atlantic salmon, a highly aerobic eurythermal species, to 12 and 20 °C, then measured cardiac mitochondrial functionality and integrity at 20 °C and at 24, 26 and 28 °C (this species’ critical thermal maximum ± 2 °C). Acclimation to 20 °C vs. 12 °C enhanced many aspects of mitochondrial respiratory capacity and efficiency up to 24 °C, and preserved outer mitochondrial membrane integrity up to 26 °C. Further, reactive oxygen species (ROS) production was dramatically decreased at all temperatures. These data suggest that salmon acclimated to ‘normal’ maximum summer temperatures are capable of surviving all but the most extreme ocean heat waves, and that there is no ‘tradeoff’ in heart mitochondrial function when Atlantic salmon are acclimated to high temperatures (i.e., increased oxidative phosphorylation does not result in heightened ROS production). This study suggests that fish species may show quite different acclimatory responses when exposed to prolonged high temperatures, and thus, susceptibility to climate warming.

## Introduction

Increases in average water temperatures and more frequent and extreme warming events (i.e., heat waves) are predicted to occur with climate change, and thus, there is an urgent need to understand the effects of prolonged and short-term warming on the physiology of aquatic organisms, including fish^1–5^. This is because the capacity of fishes to respond to changes in water temperatures is a primary driver of fish survival and distribution^6–10^, and thus, needs to be taken into account in conservation and management efforts^11^.

The response of energy metabolism to temperature stress is complex, but there is accumulating evidence that mitochondrial dysfunction plays a major role in determining ectotherm thermal tolerance (e.g., see^12–16^). The mitochondrial electron transport system (ETS) and oxidative phosphorylation are the primary processes involved in energy (ATP) production, but there are a number of temperature-related biochemical constraints that may set limits to animal performance that are associated with mitochondrial function. These include an increase in proton leak (i.e., an increase in State IV respiration, and an uncoupling of mitochondrial respiration), a loss of complex functionality (e.g., as shown for complex I, NADH dehydrogenase), an alteration in mitochondrial ADP and substrate affinities, an increase in cytochrome c, NADH and proton leak as a result of compromised mitochondrial membrane integrity (which reduces oxidative phosphorylation efficiency and disrupts mitochondrial membrane potential), and an increase in reactive oxygen species (ROS) formation (for reviews see^16,17^).

Mitochondrial properties are often highly plastic, can be adjusted during thermal acclimation or evolutionary history, and there is growing evidence that the acclimation potential of cardiac mitochondria could be key to fish thermal tolerance/adaptation^14,18–27^. However, there is limited research on how cardiac mitochondrial responses to acute heat stress are influenced by warm acclimation. Iftikar et al.^25^ acclimated New Zealand wrasse (*Notolabrus celidotus*) to mean summer temperatures, but found little to no mitochondrial benefits in the face of further climate warming. Chung et al.^20^ showed that northern and southern killifish (*Fundulus heteroclitus*) acclimated to 33 °C had decreased mitochondrial function at 38 °C compared to killifish acclimated to 15 °C. Overall, these two studies support the ‘plastic floors and concrete ceilings’ principles^28^, and suggest that mitochondria from warm-acclimated fish could have limited or reduced scope to enhance their capacity upon acute exposure to higher temperatures (i.e., climate change related heat waves) because they have already reached their maximal acclimation potential. This is important to understand as temperature-induced declines in cardiac function and aerobic metabolism are thought to limit fish performance and determine their thermal limits^2,29–31^, and mitochondrial thermal sensitivity and plasticity appear to be a major factor limiting cardiac function^14,15,32–37^.

Thus, the main objective of this study was to examine the effect of warm acclimation (i.e., to 20 °C) on the Atlantic salmon’s cardiac mitochondrial response to acute heat stress. Based on the studies of Iftikar et al.^25^ and Chung et al.^20^, we hypothesized that warm acclimation would result in very limited or no changes in mitochondrial function/properties that would improve mitochondrial capacity at higher temperatures. In addition, we wanted to assess whether the loss of cardiac mitochondrial functional capacity in Atlantic salmon corresponds with temperatures associated with cardiac collapse and the upper thermal limit of this species (see^36,38–41^). To address these questions, we assessed the functionality and integrity of isolated cardiac mitochondria from 12 °C-(cold) and 20 °C-(warm) acclimated salmon at four temperatures: 20 °C (temperature of warm acclimation), and at the salmon’s CT_Max_ (~ 26 °C) ± 2 °C (i.e., 24 and 28 °C). Then, we measured several mitochondrial bioenergetic processes underlying mitochondrial thermal sensitivity and plasticity, including: (1) the contribution of NADH dehydrogenase (complex I; CI) to mitochondrial respiration (due to its known thermal sensitivity and plasticity^32,33,35^); (2) mitochondrial respiration through the principal complexes of the electron transport system (complexes I and II; I + II); (3) mitochondrial membrane potential; (4) reactive oxygen species (ROS) production (i.e., release rate); (5) outer and inner membrane integrity; and finally (6) the maximal capacity of the ETS (ETS-I + II) and CIV to identify limiting steps in the ETS.

In these studies, we used the Atlantic salmon (*Salmo salar*, Linnaeus 1758) because it is a highly aerobic, moderately eurythermal, teleost that has substantial cardiac and metabolic capabilities for thermal adaptation^38,40,42–44^. However, the mechanisms underlying its capacity to successfully increase its aerobic scope and cardiac performance are not fully understood, notably at the mitochondrial level and in response to prolonged warming^23,36^. By studying cardiac mitochondrial physiological capacity at assay temperatures that span this species’ upper thermal window and limits, and represent the thermal profile of temperate coastal marine environments during summer^45–47^ it was hoped that these results would provide key information with regard to: (1) predicting this (and other) species’ survival and geographic distribution/expansion in an era of climate change; and: (2) understanding how physiological plasticity/capacity relates to the ability of fishes to tolerate climate-driven environmental warming and ‘heat waves’.

## Results

### Morphometric parameters

There was no difference in body mass (999.4 ± 62.8 and 1147.6 ± 62.5 g) or in relative ventricular mass (0.072 ± 0.002 and 0.073 ± 0.002) between cold- (12 °C) and warm- (20 °C) acclimated fish, respectively. There was also no difference in cardiac mitochondrial yield [i.e., the ratio of mitochondrial pellet mass to ventricular wet mass (0.039 ± 0.002 and 0.044 ± 0.002)] or in the protein content of the mitochondrial suspension (57.7 ± 2.4 mg mL^−1^ and 64.7 ± 2.6 mg mL^−1^) between cold- and warm-acclimated fish, respectively.

### Effects of heat stress on cardiac mitochondrial function

#### Respiration

**Figure 1:**
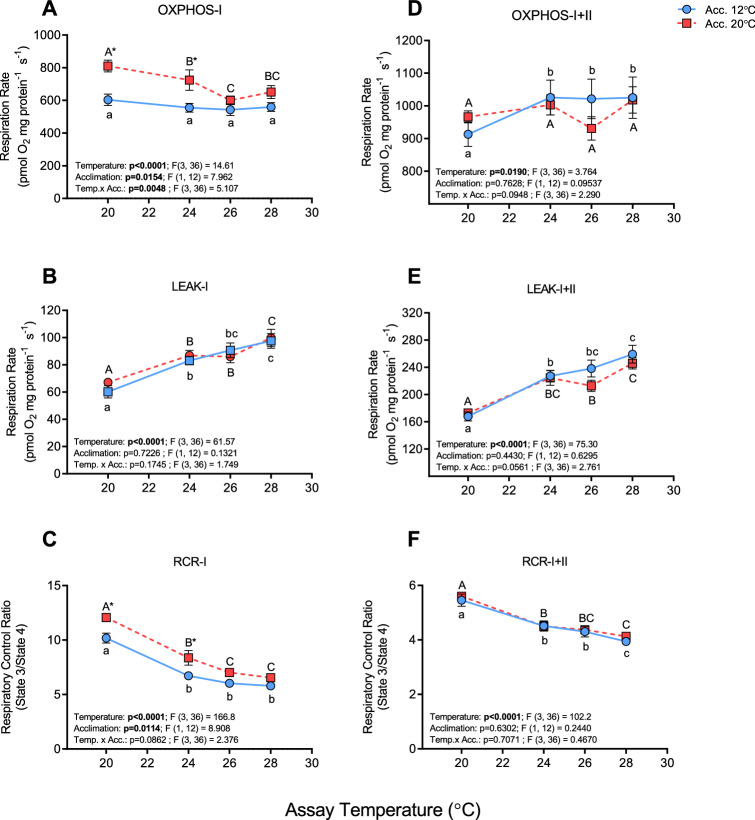
Respiration and respiratory control ratio (RCR) in cardiac mitochondria from cold- (12 °C) and warm- (20 °C) acclimated salmon when measured at 20, 24, 26 and 28 °C. Oxidative phosphorylation (OXPHOS with ADP [100 μmol L^−1^]; State 3; **A**,**D**), LEAK respiration (State 4; **B**,**E**) and the resulting RCR (State 3/State 4; **C**,**F**) were measured through complex I (**A**–**C**; with glutamate and malate as substrates) and complexes I + II (**D**–**F**; with glutamate, malate and succinate as substrates). An asterisk (*) indicates a significant (P < 0.05) difference between acclimation groups within an assay temperature, whereas letters (a, A) indicate a significant difference between assay temperatures within an acclimation group. Values are means ± s.e.m., N = 7 per group.

OXPHOS-I respiration was comparable in the cold-acclimated fish at all assay temperatures (20, 24, 26 and 28 °C). In contrast, OXPHOS-I respiration was higher at 20 °C and 24 °C in the warm-acclimated fish (by ~ 34% and ~ 30%, respectively), but not different when these mitochondria were tested at 26 and 28 °C (Fig. 1A). LEAK-I respiration increased steadily with increasing assay temperatures in both acclimation groups (by ~ 62 and ~ 48% between 20 °C and 28 °C in cold- and warm-acclimated fish, respectively; Fig. 1B). This difference in response pattern for OXPHOS-I and LEAK-I resulted in respiratory control ratio (RCR) values falling from ~ 10 and 12 at 20 °C in cold- and warm-acclimated salmon, respectively (this difference significant at 20 and 24 °C, P = 0.0038 and 0.0132, respectively), to ~ 6–7 at 26 and 28 °C (Fig. 1C). OXPHOS-I + II was ~ 25% greater at 20 °C in the two groups as compared to OXPHOS-I, but only increased from 20 to 24 °C in the cold-acclimated group, and at no point were OXPHOS-I + II values different between the two groups because CII function (as measured) was compromised by acclimation to 20 °C (Fig. 1D and Supplementary Figs. 1 and 2A). There was also no difference in Leak-I + II respiration and RCR-I + II between groups, which went from ~ 168 to 259 pmol O_2_ mg protein^-1^ s ^−1^ and from ~ 5.6 to 3.9, respectively, between 20 and 28 °C (Fig. 1E,F).

#### ROS release rate

**Figure 2:**
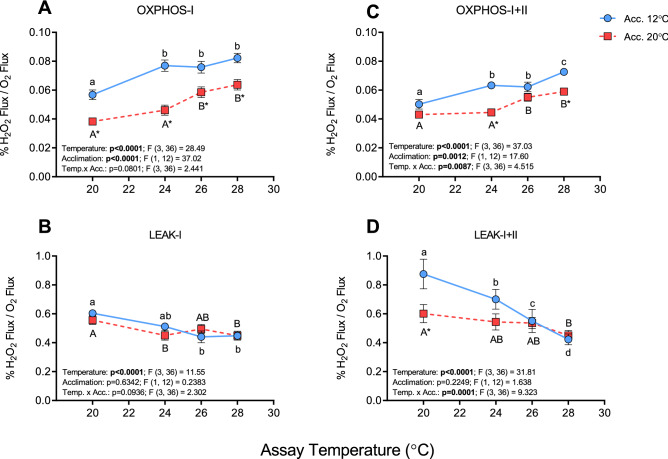
ROS release by cardiac mitochondria from cold- (12 °C) and warm- (20 °C) acclimated salmon when measured at 20, 24, 26 and 28 °C. ROS release is expressed as a percentage of O_2_ consumption (% H_2_O_2_ flux/O_2_ flux) measured during oxidative phosphorylation (OXPHOS with ADP [100 μmol L^−1^]; **A**,**C**) and LEAK (**B**,**D**) respiration through complex I (**A**,**B**; with glutamate and malate as substrates) and complexes I + II (**C**,**D**; with glutamate, malate and succinate as substrates). An asterisk (*) indicates a significant (P < 0.05) difference between acclimation groups within an assay temperature, whereas letters (a, A) indicate a significant difference between assay temperatures within an acclimation group. Values are means ± s.e.m., N = 7 per group.

ROS production (expressed as %H_2_O_2_ flux/O_2_ flux) under OXPHOS-I conditions increased in both groups as test temperature was raised from 20 to 28 °C (by ~ 44 and 68% in cold- and warm-acclimated fish, respectively) (Fig. 2A). However, warm-acclimated fish had lower %H_2_O_2_ flux/O_2_ flux under OXPHOS-I at all assay temperatures compared to the cold-acclimated fish (i.e., values were ~ 20% lower at 20, 26, and 28 °C, and ~ 40% lower at 24 °C). The results were similar for OXPHOS-I + II, although significant differences were only identified between groups at 20 °C (P = 0.0109; Supplementary Fig. 2B), 24 and 28 °C (P < 0.0001 and P = 0.0017, respectively; Fig. 2C). ROS release rate during LEAK-I respiration was not different between the two groups at any temperature, and fell slightly as test temperature increased (by ~ 25% and 20% between 20 and 28 °C in cold- and warm-acclimated fish, respectively; Fig. 2B). In contrast, ROS release rate during LEAK-I + II was ~ 45% greater at 20 °C in cold- vs. warm- acclimated salmon, and the much larger decrease in ROS release rate between 20 to 28 °C in the cold-acclimated fish (~ 50% vs. 25%) resulted in this parameter being the same in fish from both groups at 28 °C (Fig. 2D).

#### Membrane potential

**Figure 3:**
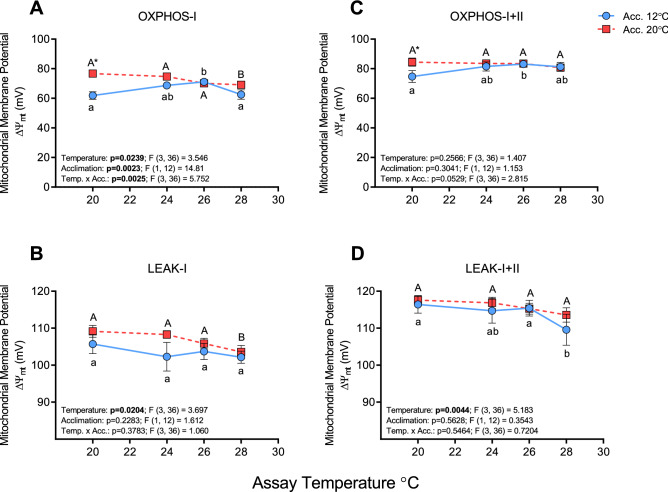
Membrane potential (ΔΨ_mt_) in cardiac mitochondria from cold- (12 °C) and warm- (20 °C) acclimated salmon when measured at 20, 24, 26 and 28 °C. Mitochondrial membrane potential (ΔΨ_mt_) was measured during oxidative phosphorylation (OXPHOS with ADP [100 μmol L^−1^]; **A**,**C**) and LEAK (**B**,**D**) respiration through complex I (**A**,**B**; with glutamate and malate as substrates) and complexes I + II (**C**,**D**; with glutamate, malate and succinate as substrates). An asterisk (*) indicates a significant (P < 0.05) difference between acclimation groups within an assay temperature, whereas letters (a, A) indicate a significant difference between assay temperatures within an acclimation group. Values are means ± s.e.m., N = 7 per group.

Mitochondrial membrane potential (ΔΨ_mt_) was ~ 10–15% higher in warm-acclimated fish (i.e., 77–84 mV vs. 66–77 mV) compared to cold-acclimated fish at 20 °C during OXPHOS-I and -I + II respiration (Fig. 3A,C). However, it was not significantly different at higher test temperatures because ΔΨ_mt_ tended to decrease slightly in the warm-acclimated fish vs. increase slightly in cold-acclimated fish (and in the presence of excess ADP, see Supplementary Fig. 2C). Membrane potential during Leak-I + II respiration was ~ 5–10 mV higher than during Leak-I respiration (i.e., ~ 117 mV vs. ~ 105–110 mV), but changed little with temperature in both groups (Fig. 3B,D).

### Membrane integrity

**Figure 4:**
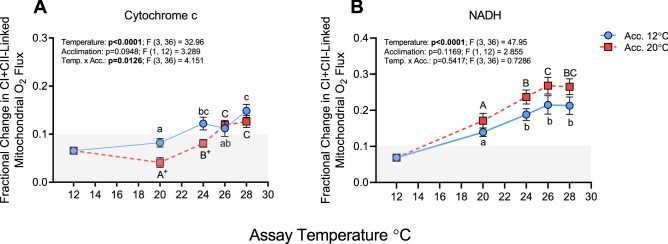
Outer and inner membrane integrity (measured as the fractional increase in respiration) in cardiac mitochondria from cold- (12 °C) and warm- (20 °C) acclimated salmon measured at 20, 24, 26 and 28 °C. Integrity of the outer (**A**) and inner (**B**) mitochondrial membranes were assessed by calculating the fractional increase in O_2_ flux following the addition of cytochrome c and NADH, respectively, during oxidative respiration (OXPHOS with excess ADP) through complexes I + II (i.e., with glutamate, malate and succinate as substrates). Control values at 12 °C for mitochondria from cold- (12 °C) acclimated fish were added in the graphs to show that the fractional increase in O_2_ flux following cytochrome c and NADH addition is normally below 0.1 (i.e., < 10%; N = 4; data not included in statistical analyses as the data were obtained using fish from a different trial). The gray area represents the range of values < 0.1. An asterisk (*) indicates a significant (P < 0.05) difference between acclimation groups within an assay temperature, whereas a plus sign (+) indicates a difference at P = 0.065, and letters (a, A) indicate a significant difference between assay temperatures within an acclimation group. Values are means ± s.e.m., N = 7 per group.

At 12 °C, the fractional increase in OXPHOS-I + II in both groups was less than 0.1 (i.e., ~ 6%) following cytochrome c (Cyt c) or NADH addition indicating that the integrity of the inner and outer mitochondrial membranes was intact (Fig. 4A, B). This was also the case at 20 °C with Cyt c for the cold-acclimated group where the fractional O_2_ flux was  ~ 8%. However, as temperature increased further, this value increased to 12–15% in this group (Fig. 4A).  In the warm-acclimated fish, the Cyt c-stimulated increase in OXPHOS-I + II was only 4% at 20 °C and ~ 7% at 24 °C, and both these values were significantly lower than measured in 12 °C-acclimated salmon (Fig. 4A). Nevertheless, the Cyt c-stimulated increase in O_2_ flux rose to ~ 12% in warm-acclimated fish at 26 °C and 28 °C. The fractional increase in respiration following NADH was already above 10% in both groups by 20 °C, and increased to ~ 20–25% by 26 °C (Fig. 4B). Although the NADH-stimulated increase in OXPHOS-I + II was consistently higher in the warm-acclimated salmon from 20–28 °C, none of these differences were significant (0.2710 > P < 0.7311; Fig. 4B).

### Maximum electron transport capacity

**Figure 5:**
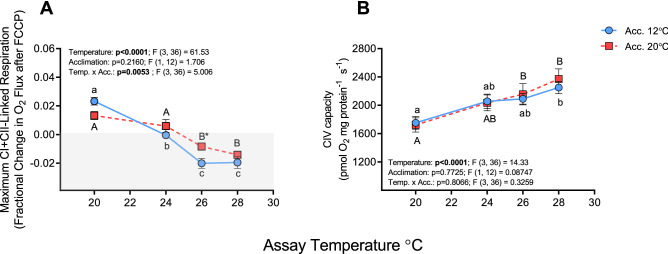
Maximum substrate oxidation capacity (ETS-I + II) and complex IV (CIV) capacity in cardiac mitochondria from cold- (12 °C) and warm- (20 °C) acclimated salmon when measured at 20, 24, 26 and 28 °C. Maximum capacity of (**A**) CI + CII-linked respiration (ETS-I + II) expressed as fractional change in O_2_ flux following addition of the uncoupler FCCP (0.25 µmol L^−1^); and of (**B**) complex IV (CIV) following the addition of the CIV electron donor TMPD (5 mmol L^−1^) + ascorbate (10 mmol L^−1^), then sodium azide (8 mmol L^−1^, an inhibitor of CIV used to correct for TMPD autoxidation). An asterisk (*) indicates a significant (P < 0.05) difference between acclimation groups within an assay temperature, whereas letters (a, A) indicate a significant difference between assay temperatures within an acclimation group. In panel A, the gray area indicates values less than zero (i.e., that FCCP had an inhibitory effect). Values are means ± s.e.m., N = 7 per group.

FCCP, added to dissipate the proton gradient, only increased respiration by ~ 1–2% over OXPHOS-I + II values in both acclimation groups at 20 °C (Fig. 5A), and this suggests that the electron transport system was already operating at maximal capacity at this temperature. Maximal respiration (ETS-I + II) was not stimulated by the addition of the uncoupler FCCP at 24 °C, and at 26 and 28 °C FCCP had an inhibitory effect and reduced ETS-I + II respiration by ~ 1–2% in both acclimation groups. In contrast, CIV capacity gradually increased with assay temperature in cold- and warm-acclimated fish (by ~ 28% and 38% between 20 and 28 °C, respectively) and was not altered by thermal acclimation (Fig. 5B).

## Discussion

Mitochondrial dysfunction may constrain whole-organism performance and survival by limiting cardiac function and setting thermal limits (see^2,13–15,20,21,32,34,36^). In the present study, we examined the thermal sensitivity and acclimation potential of Atlantic salmon cardiac mitochondrial function to provide a more comprehensive picture of how acclimation to warm temperatures affects the metabolic capacity of this highly aerobic, moderately eurythermal, fish species at temperatures just below, at, and above their CT_Max_. The importance of cardiac mitochondrial thermal plasticity for cardiorespiratory performance, and potentially in heart failure, is discussed in the context of climate change and metabolic adaption in fish^2,55^.

### Warm acclimation improves mitochondrial function at high temperatures

We have already shown that cardiac mitochondria of Atlantic salmon are thermally robust to acute and chronic exposure to 20 °C^23^. Here, we demonstrated that prolonged exposure to 20 °C: enhances cardiac mitochondrial respiration (OXPHOS-I and its RCR) at 20 and 24 °C, but not at 26 or 28 °C, as compared to 12 °C-acclimated fish (Fig. 1); and reduces ROS release rate during OXPHOS-I and/or OXPHOS-I + II from 20–28 °C, and during LEAK-I + II up to 24 °C (Fig. 2). The change in OXPHOS-I respiration is consistent with previous studies on fish heart mitochondria suggesting that thermally-induced alterations in mitochondrial respiration are linked to CI function/electron transfer capacity^32,33,35^. However, the increase in CI-dependent cardiac mitochondrial respiration in warm-acclimated fish, as compared to 12 °C-acclimated fish, at high temperatures is in direct contrast with the acclimatory response of several other fish species. Iftikar et al.^25^ reported that wrasse cardiac mitochondrial function at 20 °C was not higher in 20- vs. 15 °C-acclimated fish when measured at 20 °C. Ekström et al.^22^ showed that the catalytic capacity of CI was lower at 23–36 °C when European perch (*Perca fluvitalis*) were ‘field-acclimated’ to 22.5 vs. 15.5 °C. Finally, both Baris et al.^18^ and Chung et al.^20^ found that killifish acclimated to 28–33 °C vs. 12–15 °C had a lower OXPHOS-I respiration rate when measured at 28–38 °C. The reason(s) for these disparate results are not known. However, it is unlikely that they are related to a difference in the duration of acclimation between studies or how close the acclimation temperature was to a species’ thermal limits. While the salmon in this study and our previous study^23^ were acclimated for extended periods (62–72 and 149–163 days, respectively) as compared to the studies on killifish (4 weeks), the warm-acclimated perch used in Ekström et al.^22^ came from a population restricted to an artificially heated ecosystem in the Baltic Sea, and Pichaud et al.^27^ showed that mitochondrial function was the same in trout (*Oncorhynchus mykiss*) from 2 to 39 days after being transferred from 10–16 °C. Further, the acclimation temperature of 20 °C is approx. 3 and 6 °C below the Atlantic salmon’s incremental and critical thermal maximums (IT_max_ and CT_Max_, respectively^36,40,41^) and acclimation limit^43^, and the killifish used by Chung et al.^20^ were also acclimated close to their upper thermal limits (33 °C vs. a CT_Max_ of 41 °C and an acclimation limit of 35 °C). One potential reason that these salmon did not show a downregulation of mitochondrial respiration is that, unlike the other species, the salmon is a cold-active species with a high aerobic/metabolic capacity. It is possible that the mitochondria of these species show a different acclimatory response when exposed to temperatures approaching their thermal limits for prolonged periods as compared to species such as the wrasse, killifish and European perch. This hypothesis would be consistent with Hvas et al.^43^ who reported that salmon acclimated to 23 °C had a slightly higher maximum metabolic rate at this temperature than at 18 and 15 °C, but awaits more rigorous testing.

Interestingly, while there is clearly room for mitochondrial acclimation to high temperatures, OXPHOS-I + II was comparable between acclimation groups (Fig. 1 and Supplementary Fig. 2A). These data: suggest that CII function may have been compromised by acclimation to 20 °C (See Supplementary Fig. 1) and that there was a compensatory increase in CI function to allow maximum mitochondrial capacity to be maintained; and support previous suggestions that a loss of biochemical/cellular integrity, rather than oxygen limitation, underlies mitochondrial and cardiac dysfunction at high temperatures^14,34^. In our study, this hypothesis is supported by the reduced ROS production (Fig. 2) and preserved outer membrane integrity (Fig. 4) under OXPHOS-I + II found in warm-acclimated fish. However, the method we used to assess CII function [CII = (CI + CII)–CI] does have its limitations, and additional studies on CII function following acclimation to high temperatures, and after acute exposure to maximal temperatures, should be performed.

The increase in absolute ROS release rate (per mg protein) in 12 °C-acclimated fish when tested at 12 vs. 28 °C was ~ two-fold and three-fold during OXPHOS-I and I + II respiration (~ 0.24 vs. ~ 0.46 pmol mg protein s^-1^ and ~ 0.24 vs. ~ 0.74 pmol mg protein s^−1^, respectively; Gerber et al.^23^ vs. this study), and in both acclimation groups the relative ROS release rate (per O_2_ flux) during OXPHOS-I and I + II increased by approx. ~ 45–65% and ~ 35–45%, respectively, between 20 and 28 °C (Fig. 2 and Supplementary Fig. 2B). This increase in ROS release rate for 12 °C-acclimated fish is similar to the ~ three-fold increase reported for 15 °C-acclimated killifish when liver mitochondria were tested at 33 °C^56^, and for 10 °C-acclimated Arctic char (*Salvelinus alpinus*) heart mitochondria when measured at 25 °C (two-fold); although the char showed no increase in ROS release rate between 10 and 20 °C^32^. The most striking benefit of warm acclimation was a considerable (10–40%, depending on complex and test temperature) decrease in ROS production during OXPHOS respiration (which also increased considerably with acclimation to high temperatures), and that at 28 °C the ROS release rate was less or equal to that of 12 °C-acclimated fish at 20 °C (Fig. 2). These findings are in contrast to the results of studies on killifish acclimated to 15 vs. 33 °C^56^ and wrasse acclimated to 15 vs. 21 °C^25^, and indicate that: (1) there is no ‘tradeoff’ in mitochondrial function when Atlantic salmon are acclimated to high temperatures (i.e., increased oxidative phosphorylation does not result in heightened ROS production); and (2) warm acclimation in salmon may decrease oxidative damage related to heat stress. For example, these lower levels of ROS production may protect against myocardial oxidative stress by attenuating lipid peroxidation and damage to proteins and DNA, which are all implicated in cell damage/apoptosis/necrosis and ultimately myocardial dysfunction and heart failure^57,58^.

In this study, this decrease in ROS production does not appear to have been related to an increase in the antioxidant system as mitochondrial superoxide dismutase activity was not different between the acclimation groups^23^. Instead, it is likely that improved CI functional integrity in warm-acclimated fish (most importantly the considerable increase in RCR up to 24 °C as compared to 12 °C-acclimated fish; Fig. 1) may have played a key role in reducing ROS production at high temperatures. CI is an important site of ROS production and contributor to mitochondrial proton motive force, and the improved coupling and reduced rate of mitochondrial ROS production is consistent with the higher mitochondrial ΔΨ_mt_ observed at 20 °C in the 20 °C-acclimated fish (Fig. 3). Further, Christen et al.^32^ reported that ROS (H_2_0_2_) production was negatively related to mitochondrial respiration in Arctic char at temperatures below this species’ CT_Max_ (i.e., at 15 and 20 °C), and suggested that this was because electrons were being more efficiently channeled to complex IV of the electron transport system. In agreement, the thermal sensitivity (*Q*_*10*_) of CIV in this study tended to match that of OXPHOS-I + II in cold-acclimated fish (1.3 vs. 1.2), whereas it was higher in warm-acclimated fish (1.6 vs. 1.2). The latter suggests that a more efficient electron transfer contributed to the decreased ROS release rates observed.

The reported increase in ΔΨ_mt_ in 20 °C-acclimated salmon was not due to an increase in mitochondrial inner membrane integrity, as unlike cytochrome c (which assesses outer membrane integrity), mitochondrial O_2_ flux after the addition of NADH was not significantly different between groups at any test temperature (Fig. 4). However, we cannot rule out that differences in the amount of mitochondrial inner membrane^59^ influenced our results, or that changes in the level of mitochondrial uncoupling proteins (UCPs) played a role. Bryant et al.^60^ reported that acclimation temperature changes the mRNA expression of several UCP isoforms in the gill, brain and muscle of killifish, and Beemelmanns et al.^61^ showed that the mRNA expression of UCP-2 in the liver was downregulated by more than five-fold in salmon acclimated to 4 weeks at 20 °C vs. fish held at 12 °C. A potential role for UCP-2 in the observed differences in ΔΨ_mt_, ROS production and mitochondrial respiration is also supported by the data of Wen et al.^62^ on Chinese perch (*Siniperca chuatsi*). These authors showed that there is considerable UCP-2 mRNA expression in this species’ heart. However, Mark et al.^63^ report that skeletal muscle and liver UCP-2 mRNA and protein expression increased when Antarctic eelpout (*Pachycara brachycephalum*) were acclimated to 5 °C. Clearly, more research should be conducted on the response of cardiac UCP-2 to temperature change in fishes, and its role as a proton pore regulating mitochondrial respiration and function.

Despite the above positive changes, warm acclimation to 20 °C only appears to protect or improve mitochondrial functional capacity below an acute test temperature of 26 °C. OXPHOS-I respiration and RCR, which were enhanced by warm-acclimation at 20 and 24 °C, were not different than measured in 12 °C-acclimated fish at 26 and 28 °C (Fig. 1A,C). Furthermore, we observed several mitochondrial components that were not enhanced by thermal acclimation, and others that were negatively impacted by heat stress in the 20 °C-acclimated group. For instance, OXPHOS-I + II respiration and RCR were not different between the acclimation groups at any temperature because CII function was apparently compromised by acclimation to 20 °C (Fig. 1B and Supplementary Fig. 1). Also, while CIV capacity continued to increase at the highest temperatures tested (up to 28 °C in both acclimation groups), and ETS-I + II (i.e., after FCCP was added to dissipate the proton gradient) was still higher in 20 °C-acclimated fish at 26 °C, ETS-I + II was actually lower than OXPHOS-I + II at both 26 and 28 °C (Fig. 5). This data strongly suggests that the limiting step(s) in the ETS are upstream of CIV, and this agrees with other studies showing excess CIV capacity or activity in the fish heart^14,15,22,24,33,35^ and with Hilton et al.^33^ who showed that FCCP resulted in a slight inhibition of respiration in cardiac mitochondria from subtidal triplefins that were acutely exposed to 15, 25 or 30 °C. This inability to elevate metabolism once chemically uncoupled at high temperatures indicates that: (1) maximal mitochondrial respiration through CI and CII was reached at 20 °C; but also that (2) there is very limited ETS reserve capacity in this species (1–5%) that is reduced with rising temperatures, as suggested previously by Iftikar and Hickey^34^.

### Thermosensitivity of cardiac mitochondrial function closely matches that of whole animal thermal tolerance

Our cardiac mitochondrial data for 12 °C-acclimated Atlantic salmon fit very well with cardiorespiratory data for this species and that reported for several other fishes^32,33,64^, and suggests that limits to mitochondrial function/oxidative phosphorylation contribute to the loss of cardiac function/heart failure at high temperatures, and thus, a fish’s upper thermal tolerance. In salmon of this same strain (i.e., stocks originating from the Saint John River, New Brunswick, Canada) acclimated to 12 °C, heart rate peaks at 23–24 °C during a CT_Max_ test (at 2 °C h^−1^)^36,48^, and this temperature of maximum heart rate corresponds well with the temperature at which cardiac mitochondrial function begins to be compromised (i.e., RCR-I drops by 40% and ROS production increases by a similar amount between 20 and 24 °C, and the fractional increase in mitochondrial O_2_ flux following cytochrome c addition increases above the threshold level of 0.1 at 24 °C (Figs. 1C, 2A and 4A)). Although we have not determined the CT_Max_ of 20 °C-acclimated salmon in our lab, it is apparent in this study that acclimation to 20 °C enhances salmon cardiac mitochondrial function when acutely exposed to high temperatures (i.e., to between 24–26 °C instead of between 20–24 °C in warm- vs. cold-acclimated fish), and Anttila et al.^38^ showed that acclimation to 20 °C increased the Atlantic salmon’s temperature of maximal heart rate and onset of heart failure from ~ 21–23 °C in cold (12 °C)-acclimated to ~ 27 °C in warm (20 °C)-acclimated fish. Finally, when Atlantic salmon are acclimated to 23 °C^43^, or given an incremental temperature increase (at 1 °C week^−1^) to that temperature, mortality is only 20–30%^40^. This suggests that the mean thermal tolerance of Atlantic salmon to prolonged high temperatures is 24 °C or above, and this matches very well with the mitochondrial function data obtained in this study for 20 °C-acclimated fish.

Collectively, these data strengthen the hypothesis that links between cardiac mitochondrial thermal sensitivity, and its acclimation potential, are directly related to the thermal limit of cardiac function and a fish’s thermal tolerance^14,19,22,25,33–36^. However, a loss of mitochondrial functional capacity is clearly not the only mechanistic explanation for heart failure at high temperatures in fishes. Haverinen and Vornanen^65^ elegantly demonstrated in rainbow trout (acclimated to 12 °C) that the depression of heart rate at high temperatures (bradycardia) was due to failure of ventricular excitation (sarcolemmal ion regulation) above 25.3 °C. In agreement with Haverinen and Vornanen^65^, the increased cytochrome c and NADH flux at temperatures above 24 and 20 °C, respectively (Fig. 4), suggest that membrane fluidity and integrity in the heart is negatively impacted by high temperatures. Hence, a temperature-induced disruption of biological membranes probably triggers a cascade of failures at high temperatures. Finally, it is unclear why stroke volume does not increase at high temperatures in fish after temperature-increased bradycardia occurs to maintain or increase cardiac output (e.g. see^66^). This is very likely related to the above issue with the electrical excitability of ventricular myocytes^65^. However, the inability of mitochondria/oxidative phosphorylation to supply the ATP needed by various cellular processes, including ATP-dependent ion pumps, likely plays a significant role.

### Linking mitochondrial function and thermal tolerance in a climate change scenario

Based on a comparison of European perch from a reference site and the ‘Biotest’ site, Sandblom et al.^28^ proposed that while resting/basal cardiorespiratory functions (‘floors’) in fish are thermally plastic, maximum capacities and upper critical heat limits (‘ceilings’) are much less flexible, i.e. they are ‘concrete’. Further, they suggested that this would make fish vulnerable to the ‘heat waves’ that are predicted to accompany climate change, an interpretation that is consistent with Ekström et al.^22^ who showed that the catalytic capacity of mitochondrial complexes was reduced in ‘Biotest’ fish vs. those from the reference site at temperatures above 23 °C. However, in this study, we show that acclimation to 20 °C substantially improves the efficiency and capacity of cardiac mitochondrial respiration at 20 and 24 °C, improves mitochondrial outer membrane integrity, and that ROS production is much less in these fish from 20–28 °C, as compared to 12 °C-acclimated salmon. Given this data, and that of Anttila et al.^38^ showing that acclimation to 20 °C vs. 12 °C increased the temperature of heart failure by as much as 6 °C, it is very likely that post-smolts of this species would be able to withstand short-term temperature increases of approx. 4–5 °C (i.e., up to ≃ 24–25 °C), and thus, that most ‘heat waves’ may have limited impacts on this temperate species’ survival in coastal environments (incl. those associated with sea-cage culture) as long as other problematic abiotic (e.g., hypoxia^48^) and biotic (e.g., sea lice load^67^) factors are not present. Thus, our data suggest that some species have considerably more acclimatory capacity with regards to warm temperatures than suggested by Sandblom et al.^28^, and that the ‘plastic floors and concrete ceilings’ principles may not be universally applied to all species and situations: i.e., there are ‘cracks in the ceiling’. This may not be surprising given what 15 years of research has revealed about the applicability of the oxygen and capacity limited thermal tolerance (OCLTT) concept to fish thermal tolerance and biology (e.g., see^68^). Why the salmon appears to respond to high temperature acclimation differently (at least at the mitochondrial level) as compared to European perch and killifish is not known, but could be related to their high metabolic capacity, or that in small streams in places like New Brunswick (Canada) they can be exposed to temperatures in the summer that fluctuate daily by ~ 6–8 °C and can briefly surpass temperatures as high as 30 °C^42^.

In summary, this research has revealed a number of novel findings, and raised several important questions about intraspecific differences in temperature-dependent effects on mitochondrial physiology, this organelle’s acclimatory potential, and fish upper thermal tolerance. It is hoped that this will stimulate further research in the area of physiological processes mediating thermal tolerance/limits in fishes. For example, what mechanisms mediate inter-specific differences in mitochondrial plasticity following prolonged exposure to warm temperatures, and were responsible for the reduced ROS production in warm-acclimated Atlantic salmon despite their increased mitochondrial respiration (i.e., why was there no ‘tradeoff’?). Further, what are the implications of the latter for the redox status and oxidative damage of the heart at high temperatures^69,70^? These questions have significant implications for understanding which fish species will be the ‘winners’ and ‘losers’ with climate change.

## Methods

### Experimental animals and temperature acclimation

Atlantic salmon (*Salmo salar*) from Cape d'Or Sustainable Seafood Inc. (Advocate Harbour, Nova Scotia, Canada) were maintained and acclimated as described previously^23^. Briefly, the fish were maintained at the Laboratory for Atlantic Salmon and Climate Change Research (LASCCR, Ocean Sciences Centre, St. John’s, Newfoundland, Canada) under a 14 h light:10 h dark photoperiod and fed a commercial marine fish diet (Europa; Skretting Inc.) at ~ 1.5% body mass day^-1^. Fish were either maintained in aerated seawater at 12 °C ± 1 °C or acclimated to 20 °C by gradually increasing the temperature by ~ 0.25 °C every day until it reached this temperature. Some of the 12 °C- and 20 °C-acclimated fish were sampled between days 62 and 72 after 20 °C was reached to assess the response of this species’ cardiac mitochondria to acute and chronic warming to 20 °C^23^. The remaining fish were maintained for an additional 77 days at their acclimation temperature before sampling (experiments) to assess the mitochondrial response to heat stress (24–28 °C) after cold- (12 °C) and warm- (20 °C) acclimation. The experiments were completed within 14 days, hence salmon were thermally acclimated between 149 and 163 days to 12 or 20 °C.

All experimental procedures followed guidelines established by the Canadian Council on Animal Care and were approved by the Institutional Animal Care Committee of Memorial University of Newfoundland (Protocol ^#^16–92-kg).

### Mitochondrial isolation

Cardiac mitochondria were isolated as described previously^23^. Briefly, fish were killed by a blow to the head before fork length and body mass were taken. Then, the ventricle was dissected out and left to beat in ice-cold isolation media (in mmol L^−1^: 230 mannitol; 75 sucrose; 20 HEPES; 1 EGTA, pH 7.4) for a minute before being cut in half, rinsed, blotted dry and weighed. The ventricle was then thoroughly minced and gently homogenized in three volumes of isolation media using an ice-cooled glass-homogenizer and six passes of a loose-fitting motor driven Teflon pestle. The crude homogenate was centrifuged at 800 *g* for 10 min at 4 °C to remove cell debris, and the resulting supernatant was centrifuged at 8000 *g* for 10 min at 4 °C to pellet the mitochondria. The mitochondrial pellet was washed twice by gentle re-suspension in ice-cold isolation medium containing 10 mg mL^−1^ of BSA (fatty acid free; Sigma-Aldrich, CAS 9048–46-8) and centrifugation at 8000 *g* for 10 min at 4 °C. The final pellet was weighed, and then gently re-suspended in four volumes of ice-cold respiration medium [in mmol L^−1^:160 KCl; 30 HEPES; 10 KH_2_PO_4_; 1 EGTA; 10 mg mL^−1^ BSA (fatty acid free), pH 7.4]. The protein content of the mitochondrial suspensions was determined using the Bradford assay with BSA as a standard (Thermo Fisher Scientific, Waltham, MA, USA) and respiration medium with BSA as a blank. Mitochondrial suspensions were kept on ice for 30 min to rest before use*.*

### Experimental protocols

#### Mitochondrial physiology

The function of isolated cardiac mitochondria from 12 °C- and 20 °C-acclimated salmon was assessed over a range of temperatures (20–28 °C) at which critical physiological limits in cardiac and respiratory rates of Atlantic salmon have been reported^36,38,41,48^. Our experimental protocol is described in detail below and shown in Supplementary Fig. 3.

#### Measurement of coupled mitochondrial respiration, ROS production and mitochondrial membrane potential

Mitochondria (0.125 mg protein mL^−1^) from each fish were added to 2 mL of 100% air-equilibrated respiration medium at 20, 24, 26 or 28 °C. A saturating concentration of complex I (CI) substrates, glutamate (15 mmol L^−1^) and malate (2 mmol L^−1^), and ADP (100 μmol L^−1^) were then added to measure maximal oxidative phosphorylation (OXPHOS, State 3 respiration) via complex I (OXPHOS-I). After the depletion of ADP, leak respiration (State 4) via CI (LEAK-I) was measured. Thereafter, a saturating concentration of substrate for CII (succinate, 5 mmol L^−1^) was added followed by ADP (100 μmol L^−1^) to measure maximal OXPHOS respiration (State 3) via CI + II (OXPHOS-I + II). After the depletion of ADP, leak respiration rate (State 4) via CI + CII (LEAK-I + II) was measured. Then, an excess amount of ADP (800 μmol L^−1^) was added. When ADP-stimulated respiration plateaued at a maximum level we also assessed: (1) the integrity of the inner and outer mitochondrial membranes by adding cytochrome c (10 μmol L^−1^) and NADH (0.5 mmol L^−1^), respectively; (2) the capacity of CI + CII for electron transport (ETS-I + II) using FCCP as an uncoupler; and (3) the capacity of complex IV (CIV) for electron transport by adding TMPD (0.5 mmol L^−1^) and ascorbate (10 mmol L^−1^). Sodium azide (8 mmol L^−1^) was then added to correct for TMPD auto-oxidation and terminate the experiment. All parameters were recorded in real-time using DatLab v 7.3 software (Oroboros Instruments, Innsbruck, Austria).

Mitochondrial respiration was measured using high-resolution respirometry (O2k-polarographic O_2_ sensor, Oroboros Instruments, Innsbruck, Austria) following standard operating procedures^49,50^. Recommended oxygen solubility factors (FM) for KCl experimental medium at 20, 24, 26 and 28 °C were used for accurate oxygen concentration measurements using published O_2_ solubilities^51^. ROS production was estimated by measuring extra-mitochondrial H_2_O_2_ using Amplex UltraRed (AmR, 10 µmol L^−1^), horseradish peroxidase (HRP, 3U mL^−1^) and SOD (25U mL^−1^). The ROS signal was calibrated by the addition of H_2_O_2_ (0.1 μmol L^−1^) and detected using the Green Fluorescence-Sensor of the O2k-Fluo LED2-Module (with gain and LED intensity set to 1000 and 500 mV, respectively) following standard operating procedures^52,53^. Mitochondrial membrane potential (Δψmt) was assessed using the O2k-TPP^+^ ISE-Module. The TPP^+^ selective electrodes were calibrated by incremental additions of TPP^+^ following standard operating procedures^54^ and Δψmt was estimated from TPP^+^ concentration ([TPP^+^]) in the chamber, using a modified Nernst Eq. (1):

1 $${ \Delta \Psi }_{mt}=\frac{a\,\mathrm{log }({[TPP}^{+}]{}_{added} - {[TPP}^{+}]{}_{external})\, (b)}{\left(V\right)\left(C\right)({[TPP}^{+}]{}_{external})}$$

where, a = RT/F, R is the universal gas constant (8.314); T is the absolute temperature in Kelvin; F is the Faraday constant (9.65 × 10^4^); added and external [TPP^+^] are the concentration of TPP^+^ measured in the O2k-chamber after addition of TPP^+^ during the calibration process and at any specific time after addition of substrates, respectively; b is the binding constant of TPP^+^ (0.16; to correct for non-specific binding of TPP^+^); v is the mitochondrial matrix volume (0.0025 mL mg^−1^ protein) and C is the mitochondrial concentration (mg protein ml^−1^).

#### Calculation of mitochondrial respiration coupling, efficiency and integrity

Respiratory control ratios [RCR; i.e. State 3/State 4] in the presence of CI and CI + II substrates were calculated to assess the mitochondrial coupling efficiency. The fractional change in mitochondrial CI + CII-linked respiration stimulated by exogenous cytochrome c, NADH and FCCP [i.e. (CI + CII OXPHOS_stimulated_ − CI + CII OXPHOS)/CI + CII OXPHOS_stimulated_] was used as an index for outer mitochondrial membrane integrity, inner mitochondrial membrane integrity and CI + CII maximum mitochondrial capacity in a non-coupled state (ETS-I + II), respectively.

### Statistical analyses

Statistical analyses were performed using GraphPad Prism 8 software (La Jolla, CA, USA). Effects of acclimation (12 vs. 20 °C) and assay temperature (20, 24, 26 and 28 °C) on all mitochondrial parameters were assessed using repeated measured two-way ANOVAs followed by Sidak’s multiple comparisons tests. The morphometric data were analyzed using unpaired two-tailed t-tests. The level of statistical significance for all analyses was P < 0.05.

## Supplementary Information

Supplementary Information

## Data Availability

The datasets generated during the current study are available from the corresponding author on reasonable request.
