# Supplementary material for: Improved mitochondrial function in salmon (Salmo salar) following high temperature acclimation suggests that there are cracks in the proverbial ‘ceiling’
Source: Sci Rep. 2020 Dec 10;10:21636. doi: 10.1038/s41598-020-78519-4 (PMC7729908; doi:10.1038/s41598-020-78519-4)
Supplement: Supplementary file 1 — Supplementary Information [file 41598_2020_78519_MOESM1_ESM.docx]

**Supplementary Figures**

**Supplementary Figure 1: Estimated Complex II dependent-respiration in cardiac mitochondria from cold- (12°C) and warm- (20°C) acclimated salmon when tested at 20, 24, 26 and 28^o^C.** Calculated by subtracting mitochondrial OXPHOS-I from mitochondrial OXPHOS-I+II respiration. An asterisk (*) indicates a significant (P < 0.05) difference between acclimation groups within an assay temperature, whereas letters (a*,* A) indicate a significant difference between assay temperatures within an acclimation group. Values are means + s.e.m., N = 7 per group.

**Supplementary Figure 2: Respiration (A), ROS production (B) and membrane potential (C) measured in cardiac mitochondria from cold- (12°C) and warm- (20°C) acclimated salmon when tested at 20, 24, 26 and 28^o^C and with an excess amount of ADP 800 µM.** An asterisk (*) indicates a significant (P < 0.05) difference between acclimation groups within an assay temperature, whereas letters (a*,* A) indicate a significant difference between assay temperatures within an acclimation group. Values are means + s.e.m., N = 7 per group.


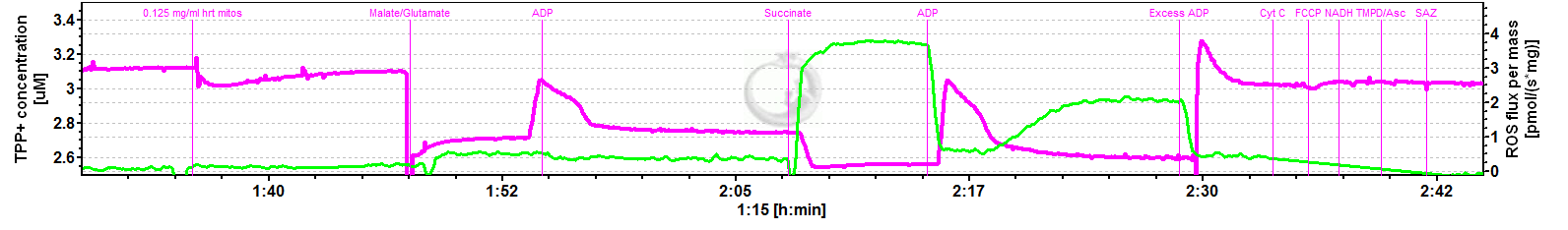

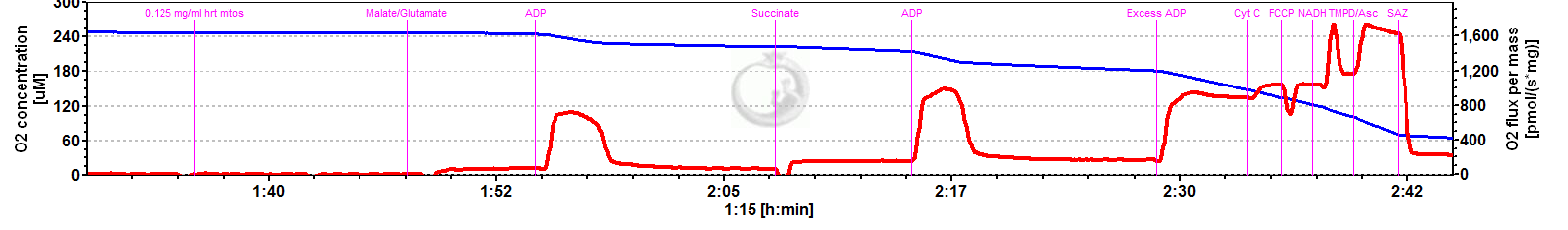
**Supplementary Figure 3:** **Representative recordings made on cardiac mitochondria (0.125 mg protein mL^-1^) from cold- (12°C) acclimated fish incubated at 20°C.** The figure shows the simultaneous measurement of mitochondrial O_2_ consumption, ROS production and membrane potential in the presence of substrates of complex I alone [malate (2 mmol L^-1^) and glutamate (15 mmol L^-1^)] and complexes I and II [+ succinate 5 mmol L^-1^] during OXPHOS (State 3) and LEAK (State 4) respiration [with 100 and 800 μmol L^-1^ ADP]. The integrity of the outer and inner mitochondrial membranes was determined using cytochrome c (Cyt c; 10 µmol L^-1^) and NADH (0.5 mmol L^-1^). Maximal respiration capacity was assessed after addition of the uncoupler FCCP (0.25 µmol L^-1^). The capacity of CIV was determined using the CIV electron donor TMPD (5 mmol L^-1^) + ascorbate (Asc; 10 mmol L^-1^) – sodium azide (SAZ; 8 mmol L^-1^, an inhibitor of CIV used to correct for TMPD autoxidation). O_2_ concentration in the chamber (in μmol L^-1^) is represented by the blue line, O_2_ flux [i.e., mitochondrial respiration rate (in pmol O_2_ (s*mg)^-1^)] by the red line, H_2_O_2_ flux [i.e., mitochondrial ROS production (in pmol H_2_O_2_ (s*mg)^-1^)] by the green line, and TPP^+^ concentration [i.e., mitochondrial membrane potential (in µmol L^-1^ of TPP^+^)] by the purple line.
